# Traumatic Brain Injury Induces Tau Aggregation and Spreading

**DOI:** 10.1089/neu.2018.6348

**Published:** 2019-12-11

**Authors:** George Edwards, Jing Zhao, Pramod K. Dash, Claudio Soto, Ines Moreno-Gonzalez

**Affiliations:** ^1^ Mitchell Center for Alzheimer's Disease and Related Brain Disorders, Department of Neurology, The University of Texas Health Science Center at Houston, Houston, Texas.; ^2^ Department of Neurobiology and Anatomy, The University of Texas Health Science Center at Houston, Houston, Texas.; ^3^ Department of Cell Biology, Networking Research Center on Neurodegenerative Diseases (CIBERNED), Facultad Ciencias, Universidad de Malaga, Malaga, Spain.

**Keywords:** Alzheimer disease, chronic traumatic encephalopathy, risk factor, tau aggregation, traumatic brain injury

## Abstract

The misfolding and aggregation of tau protein into neurofibrillary tangles is the main underlying hallmark of tauopathies. Most tauopathies have a sporadic origin and can be associated with multiple risk factors. Traumatic brain injury (TBI) has been suggested as a risk factor for tauopathies by triggering disease onset and facilitating its progression. Several studies indicate that TBI seems to be a risk factor to development of Alzheimer disease and chronic traumatic encephalopathy, because there is a relationship of TBI severity and propensity to development of these illnesses. In this study, we evaluated whether moderate to severe TBI can trigger the initial formation of pathological tau that would induce the development of the pathology throughout the brain. To this end, we subjected tau transgenic mice to TBI and assessed tau phosphorylation and aggregation pattern to create a spatial heat map of tau deposition and spreading in the brain. Our results suggest that brain injured tau transgenic mice have an accelerated tau pathology in different brain regions that increases over time compared with sham mice. The appearance of pathological tau occurs in regions distant to the injury area that are connected synaptically, suggesting dissemination of tau aggregates. Overall, this work posits TBI as a risk factor for tauopathies through the induction of tau hyperphosphorylation and aggregation.

## Introduction

Tau is a microtubule associated binding protein that, under normal physiological conditions, provides cytoskeletal support allowing axonal transport, among other functions. Tau undergoes post-translational modifications, such as phosphorylation, necessary for its regular function. Abnormal phosphorylation, however, triggers microtubule-bound tau to be released.^1,2^ Hyperphosphorylated tau (ptau) aggregates generate neurofibrillary tangles (NFTs) that are considered the pathological hallmark of tauopathies including Alzheimer disease (AD), chronic traumatic encephalopathy (CTE), and frontal-temporal dementia (FTD), among others.^2–5^

In these tauopathies, tau follows an explicit and predictable spatiotemporal pattern of deposition. Neuropathological Braak staging of tau accumulation in AD progresses from transentorhinal/locus coeruleus (stages I/II), limbic (stages III/IV), and isocortical (stages V/VI).^6–8^ In CTE, tau is found around small vessels at the depths of sulci in the cerebral cortex (stage I), superficial layers of adjacent cortex (stage II), frontal, insular, temporal and parietal cortices, and amygdala, hippocampus, and entorhinal cortex (stage III), and finally ptau pathology is found widespread in the entire brain (stage IV).^9^

Approximately 10 million traumatic brain injury (TBI) cases are reported worldwide each year.^10,11^ A moderate to severe TBI, commonly seen in car accidents, will produce systemic complications after the insult and can create long-term adverse effects in the brain or even death.^12–14^ The primary insult of a TBI can trigger systemic alterations in the brain as well as initiate secondary mechanisms in the form of multiple molecular and cellular deviations perturbing the overall environment of the central nervous system.^13–15^

Epidemiological studies indicate TBI is a risk factor for tauopathies.^15–18^ After a TBI event, ptau and NFTs can be detected as early as 6 h.^19,20^ In fact, elevated NFTs levels have been found in approximately one third of individuals post-mortem who had a surviving moderate to severe TBI demonstrating the relationship between tau aggregation and a single TBI.^21^ In addition, the diagnosis of dementia was significantly increased up to 30 years after TBI with the strongest association with TBI severity.^19^ More so, the greater the severity of TBI increases the risk of AD.^11,12,15,22^ The risk of CTE is also suggested to be increased when linked to the number of TBI events and the length of time contact sports athletes and military personnel are active.^23,24^

Recently, tau aggregates have been attributed with prion-like properties, including *in vitro* and *in vivo* seeding and spreading capability.^25–29^ Addition of a pre-formed tau seed can accelerate misfolding and aggregation of monomeric tau *in vitro* as well as exacerbate endogenous tau aggregation and deposition in the brain of mice.^30–37^ In pathological conditions, ptau can mislocalize from the axon to the soma and dendrites and can be released from the neurons.^38,39^ It has been suggested that spreading can be because of tau propagating by the functional connectome in the brain.^40–42^ Inoculation of synthetic pre-formed fibrils or tau seed-containing brain homogenate in different brain regions induces further tau accumulation downstream to their efferent areas.^40–42^ The etiology of many tauopathies is mostly sporadic.^2,4,43,44^

Several studies have identified a long list of risk factors leading to pathology. The specific event that triggers the formation of misfolded aggregates remains uncertain, however. The formation of tau misfolded aggregates could be a result of TBI events. To address this, we have induced TBI in a mouse model of tau aggregation and analyzed its effect on the severity of the clinical signs and tau spreading and deposition.

## Methods

### Animals

P301S transgenic (Tg) mice (Prnp-MAPT*P301S P301SVle/J, Jackson Laboratory, Bar Harbor, ME) express the P301S mutant human microtubule-associated protein tau (MAPT) under the control of the mouse prion protein (Prnp) promoter. These animals accumulate ptau, and NFT formation can be detected at approximately 6–9 months of age.^45^ The expression of the mutant human MAPT is five-fold higher than the expression of the endogenous mouse MAPT.

Animals were housed in groups of up to five in individually ventilated cages under standard conditions (22°C, 12h light–dark cycle) receiving food and water *ad libitum.* All animal experiments were performed in accordance with the National Institutes of Health (NIH) regulations and approved by the committee of animal use for research at the University of Texas Health Science Center at Houston, McGovern Medical School.

### Induction of TBI

Groups of 3-month-old P301S mice and wild-type (WT) littermates were anesthetized with isoflurane, placed on a stereotaxic apparatus with a face mask, and prepared for controlled cortical impact (CCI), as performed previously to induce moderate to severe TBI.^15,46,47^ Briefly, a midline incision was made, and a 5-mm–diameter craniotomy was performed on the right parietal cortex, midway between the bregma and the lambda with the medial edge of the craniotomy 1.0 mm lateral to the midline. A pneumatic impact device (Custom Design and Fabrication, Richmond, VA) was used to deliver an impact at 3.0 m/sec generating a 1.0 mm deformation on the posterior cortex.

The incision was closed, and animals were assessed in latency of foot, tail, and righting reflexes as previously described (Supplementary Fig. 1A; see online supplementary material at www.liebertpub.com).^48^ Animals were allowed to completely recover in a warm chamber before being returned to their home cages. Sham-operated animals received all the analogous surgical procedures except for the craniotomy and impact. Animals were monitored thereafter daily for up to two weeks. The TBI and sham groups were *n* = 4–6 at each time point and were selected randomly and placed in different time-point groups.

### Behavioral analysis

Barnes maze was performed six months after CCI in both groups (Tg and WT) as well as in the sham group as described previously.^49^ Barnes maze is a medial temporal lobe dependent task that evaluates spatial learning and memory. Barnes maze consists of a circular platform with 40 holes with one being an exit from the arena, and the animal uses spatial cues to discover the stationary escape hole in a 3 min trial. Mice were trained on day 1 with two acquisition trials and then four trials per day for four days. At seven days later, long-term memory was assessed.

Primary latency to the escape hole was used to assess learning and memory. Task performance was recorded and analyzed using the TopScan 2.0 tracking software (Clever Sys, Reston, VA). Behavioral analysis was performed by a single experimenter blinded to subject identities.

### Immunohistochemistry

The TBI and sham mice were sacrificed either one day, one week, 1–2 months, or six months after CCI trauma. Mice were sacrificed by CO_2_ inhalation and perfused transcardially with phosphate buffered saline (PBS). Brains were removed, post-fixed into 10% neutral buffered formalin fixative solution, and embedded in paraffin. Paraffin-fixed brain tissue was sliced coronally by microtome obtaining 10-μm–thick serial sections; these were processed for immunostaining, as reported previously.^50–52^

After blocking the endogenous peroxidase activity with 3% H_2_O_2_-10% methanol for 20 min, sections were incubated overnight at room temperature in monoclonal AT8 phospho-PHF-tau pSer202+Thr205 antibody (1:100, Thermo Fischer). Primary antibody was detected by incubating 1 h with sheep antimouse horseradish peroxidase-linked secondary antibody (General Electric, Pittsburgh, PA), and peroxidase reaction was visualized using DAB Kit (Vector, Burlingame, CA) following the manufacturer's instructions. Finally, all sections were dehydrated in graded ethanol, cleared in xylene, and cover-slipped with DPX mounting medium.

### Immunohistochemistry and tau burden quantification

Pathological tau burden was quantified using AT8 antibody assessing the overall, ipsilateral (Ipsi) and contralateral (Contra) side of CCI impact of the cortex and amygdala (Ctx), hippocampal area (Hp), and brainstem (BS). Coronal areas assessed were based on anatomical connectivity and cytoarchitectural patterns. The area rostral to the impact covered interaural (IL) ±4.54 mm, bregma (B) ±0.74 mm – IL ±3.46 mm, B ± -0.34 mm; the rostral impacted area was assessed from IL ±2.74 mm, B ± -1.06 mm to IL ±1.50 mm, B ± -2.30 mm; the caudal impacted area included IL ±1.34 mm, B ± -2.46 mm – IL ±0.00 mm, B ± -3.80 mm; whereas the caudal area to the impact was analyzed between IL ± -1.16 mm, B ± -4.96 mm and IL ± -3.16 mm, B ± -6.96 mm.

Three to five sample slides of every 10th section were examined under a bright field microscope (DMI6000B, Leica Microsystems, Buffalo Grove, IL), and photomicrographs were taken with a digital camera (DFC310 FX Leica), imported into ImageJ 1.45s software (NIH), and converted to black and white images. Threshold intensity was used to quantify AT8 burden in the brain. Threshold intensity was adjusted automatically to remove the background and held constant during quantification of all the analyzed animals.

Burden was defined as the immune-reactive area per total area analyzed. Likewise, AT8 assessment was measured in 5–7 slides of every 10th section and scored from no tau detected (0 score) to highest tau deposition (5 score). This semi-quantitative score was graded by the volume and abundance of AT8 immunoreactivity of disease-associated morphological tau adapted previously.^37,40,53^ Score values were averaged and imported into a custom-designed heat map software to generate an overall view of tau deposition. The experimenter was blinded to the animal identities and groups during processing and subsequent quantification methods.

### Enzyme-linked immunosorbent assay (ELISA) quantification

Additional 3-month-old mice (*n* = 3–4) were CCI-induced or sham-induced. The TBI and sham mice were sacrificed after one day. The ipsilateral and contralateral sides of the brains were extracted from injured and sham mice. Brain hemispheres were homogenized at 10% w/v in PBS containing protease inhibitors (cOmplete™ Protease Inhibitor Cocktail, MilliporeSigma, Bedford, MA) as described previously.^54,55^

Brain homogenates were centrifuged at 32,600 rpm for 1 h at 4°C in an ultracentrifuge (Beckman-Coulter). The supernatant was removed, and pellets were resuspended in 200 μL of 70% formic acid followed by sonication. Samples were centrifuged for 30 min under the same conditions, and the supernatant was collected and neutralized in 1 M Tris-HCl buffer, pH 10.8 to measure the fraction of insoluble tau in brain after TBI. Levels of phosphorylated tau at Ser199 were measured using Human tau [pS199] ELISA kit (Invitrogen) per manufacturer's instructions on an ELISA plate reader (EL800 BIOTEK).

### Statistical analysis

Graphs are expressed as means ± standard error of the mean (SEM). Data were tested for normal distribution by Shapiro-Wilk normality test. Student *t* test was used to analyze normal data for AT8 burden quantifications as well as non-parametric data were run with the Mann-Whitney test. Two-way analysis of variance (ANOVA) followed by a *post hoc* Tukey multiple comparisons test and Bonferroni post-test were used to analyze the Barnes maze learning curve, and one-way ANOVA was used for long-term memory. Statistical analyses were performed using GraphPad Prism 5.0 software (GraphPad Software Inc., La Jolla, CA). Statistical differences for all tests were considered significant at the *p* < 0.05 level.

## Results

### TBI induces rapid acceleration of tau hyperphosphorylation

In an effort to determine the effect of TBI in the induction of tau aggregates, 3-month-old P301S Tg mice were subjected to TBI by CCI and sacrificed one day, one week, two months, or six months post-injury. A set of P301S Tg mice was sham-induced (skin opened under anesthesia) and used as a control. To assess the effect of CCI injury and associative degree of tau pathology on behavior, acute neurological assessment was performed after CCI.

Animals exposed to TBI had impairments in simple, non-postural somatosensory functions and complex, postural somatosensory functions (*p* < 0.001) indicating the immediate impact of CCI injury on the central nervous system (Supplementary Fig. 1A; see online supplementary material at www.liebertpub.com). Hematoma and edema were seen transiently after TBI in brain at one day and one week post-operation. Later time points revealed a collapsed deformation (Supplementary Fig. 1B).

Brains were analyzed by immunohistochemistry to determine the presence of ptau using AT8 antibody. We assessed burden quantification of the immunoreactive AT8-positive area and generated an overall heat map of tau deposition in brain in both TBI-induced mice and sham animals from early to late stage time points. The presence of AT8-positive tau aggregates was analyzed both in the ipsilateral and contralateral hemispheres and in four brain areas (rostral to the impact, in the rostral impacted area, in the caudal impacted area, and caudal to the impact).

**FIG. 1.**
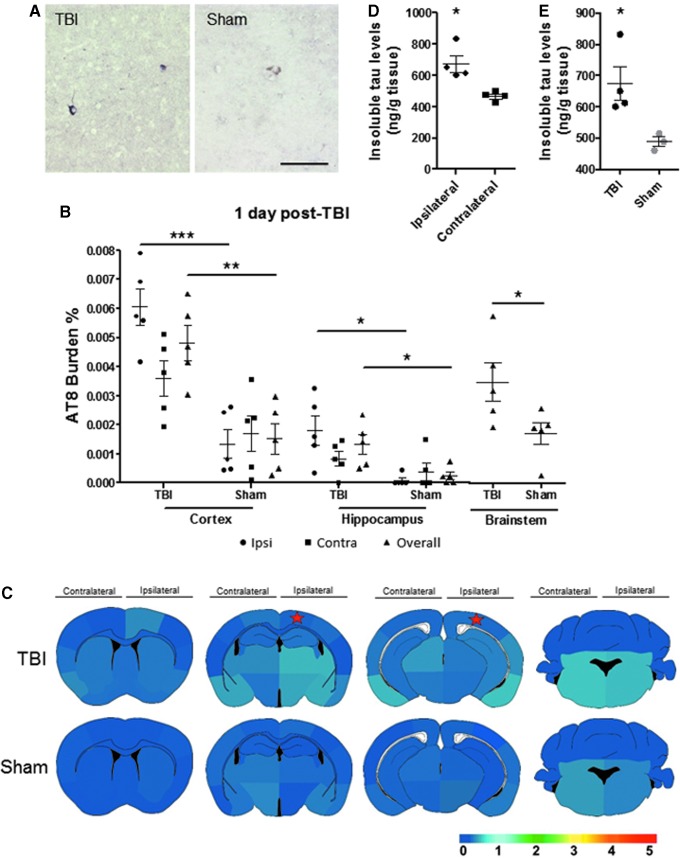
Tau aggregation is induced one day after traumatic brain injury (TBI). Tau hyperphosphorylation was assessed in brain homogenates and coronal tissue sections in three-month-old P301S TBI and sham induced mice one day after the event. (**A**) Representative bright field microscopy images of AT8 immunoreactivity in TBI and sham mice brain sections. (**B**) The AT8 burden quantification was obtained by analyzing the percentage of the area reactive with AT8 antibody in relation to the total area analyzed in the cortex/amygdala, hippocampal area, and brainstem. Statistical analysis was performed by Student *t* test or Mann-Whitney test for the ipsilateral (Ipsi) and contralateral (Contra) areas of impact as well as overall brain region. Ipsilateral and contralateral hippocampus quantifications were analyzed by Mann-Whitney test. (**C**) Semi-quantitative evaluation (lowest [0] to highest [5]) of tau deposition is captured in the heat map at different distance from the impacted area. The star denotes the estimated placement of impact. (**D,E**) Insoluble tau levels were evaluated by serial extraction and enzyme-linked immunosorbent assay performed in the formic acid fraction of the brains. Graphs in panels B, D, and E show the mean ± standard error of the mean of the *n* = 5 animals analyzed per group. Scale bar: 100 μm. *p < 0.05, **p < 0.01, ***p < 0.001.

Just one day after the TBI induction, we detected pathological tau in P301S TBI mice in comparison with age-matched sham mice in primarily the overall and Ipsi area of the impacted side (Fig. 1A,B). We observed a significant difference of AT8 burden (percentage of AT8-immunoreactive area per total area analyzed) in the Ctx, Hp, and BS (Ipsi Ctx p < 0.001; overall Ctx *p* < 0.01, overall Hp, Ipsi Hp, and BS *p* < 0.05) (Fig. 1B).

With these data, we created the spatial heat map of tau deposition in the brain of TBI versus sham mice (Fig. 1C). In P301S mice subjected to TBI, AT8-immunoreactive tau developed highest in the amygdala, in particular lateral and medial amygdaloid nucleus; neocortical areas such as entorhinal, piriform, and perirhinal cortices; as well as brainstem structures, such as pons and medulla. The deposition exhibited a range from a caudal to rostral mosaic and ventral to dorsal approach within and near the area of impact as well as below the impact, mainly affecting the hippocampal formation.

To determine the aggregation stage of the phosphorylated tau, we performed an ELISA test to detect the amount of insoluble tau. Brain homogenates from a different set of animals were ultra-centrifuged to obtain the formic acid-soluble fraction. The TBI-induced animals had higher levels of insoluble tau in the ipsilateral hemisphere than the non-impacted area (Fig. 1D). In addition, the level of insoluble tau was increased in the TBI group compared with sham mice (Fig. 1E).

**FIG. 2.**
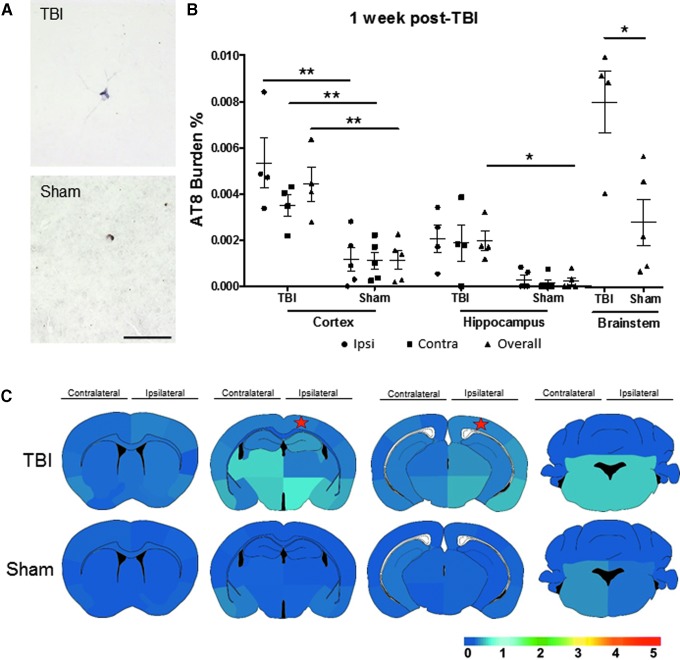
Spatial pattern and burden of pathological tau one week after traumatic brain injury (TBI). The P301S mice having either a TBI or sham surgery were assessed for tau aggregate formation and spreading by AT8 immunostaining. (**A**) Representative images of the TBI and sham animals using AT8 antibody are displayed. (**B**) The AT8 burden was evaluated in the cortex, hippocampal area, and brainstem in overall, ipsilateral (Ipsi), and contralateral (Contra) side of impact. Ipsilateral and contralateral hippocampus were evaluated by Mann-Whitney test, while all other data underwent Student *t* test. (**C**) Four coronal areas per animal were assessed to re-create a heat map to navigate tau spreading after TBI. Heat map scale: lowest (0) to highest (5) score. The star denotes the estimated placement of impact. Scale bar: 100 μm. *p < 0.05, **p < 0.01.

To evaluate the AT8 burden levels over time, we analyzed tau deposition in TBI and sham mice after one week. Immunohistochemical staining using AT8 antibody (Fig. 2A) was quantified in both groups. The TBI mice displayed a significantly exacerbated tau pathology compared with sham mice one week post-injury in the following brain regions (Ipsi, Contra, and overall Ctx *p* < 0.01; overall Hp and BS *p* < 0.05) (Fig. 2B).

**FIG. 3.**
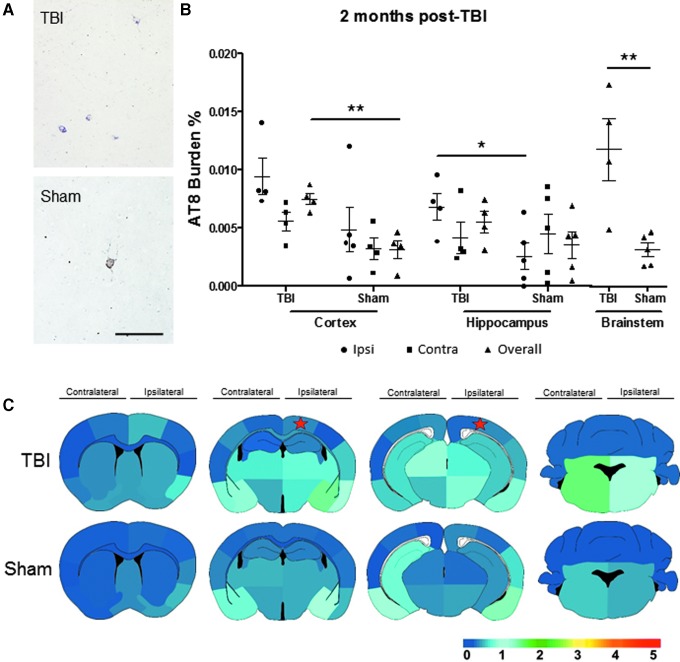
Tau aggregation is significantly increased two months after traumatic brain injury (TBI) injury. (**A**) Illustrative microphotography of AT8 immunopositive staining visually displayed growing pathologic tau aggregation. (**B**) The AT8 burden quantification in the overall, ipsilateral (Ipsi), and contralateral (Contra) impacted area in the three main brain regions analyzed. Ipsilateral cortex and Contra hippocampus were evaluated by Mann-Whitney test, while all other statistical analysis was performed by Student *t* test. (**C**) The spatial heat map revealed increasing tau burden and presence of pathogenic tau aggregates in multiple areas in TBI-induced mice having more aggressive pathology compared with sham mice. Scale bar: 100 μm. Heat map scale: lowest (0) to highest (5) score. The star denotes the estimated placement of impact. *p < 0.05, **p < 0.01.

The spatial heat maps revealed more sporadic tau formation in the TBI mice in comparison with their respective sham counterparts (Fig. 2C). We observed that after one week, TBI mice projected a spatial tau pattern reminiscent of the one day TBI mice, but extending to the contralateral side of impact of the cortical and hippocampal areas, albeit at modest yet progressing levels, and increased compared with age-matched sham mice. At 1–2 months after CCI induction, TBI mice demonstrated higher AT8 burden in the overall Ctx and BS (*p* < 0.01) and Ipsi Hp (*p* < 0.05) as observed in the representative pictures and the burden quantification **(**Fig. 3A,B). Both groups represented advancing tau pathology at 4–5 months of age; however, TBI-induced animals demonstrated higher average burden scores in comparison with sham mice as observed in the heat map of global tau distribution (Fig. 3C).

### TBI induces long-term cognitive impairment and accelerated tau pathology.

**FIG. 4.**
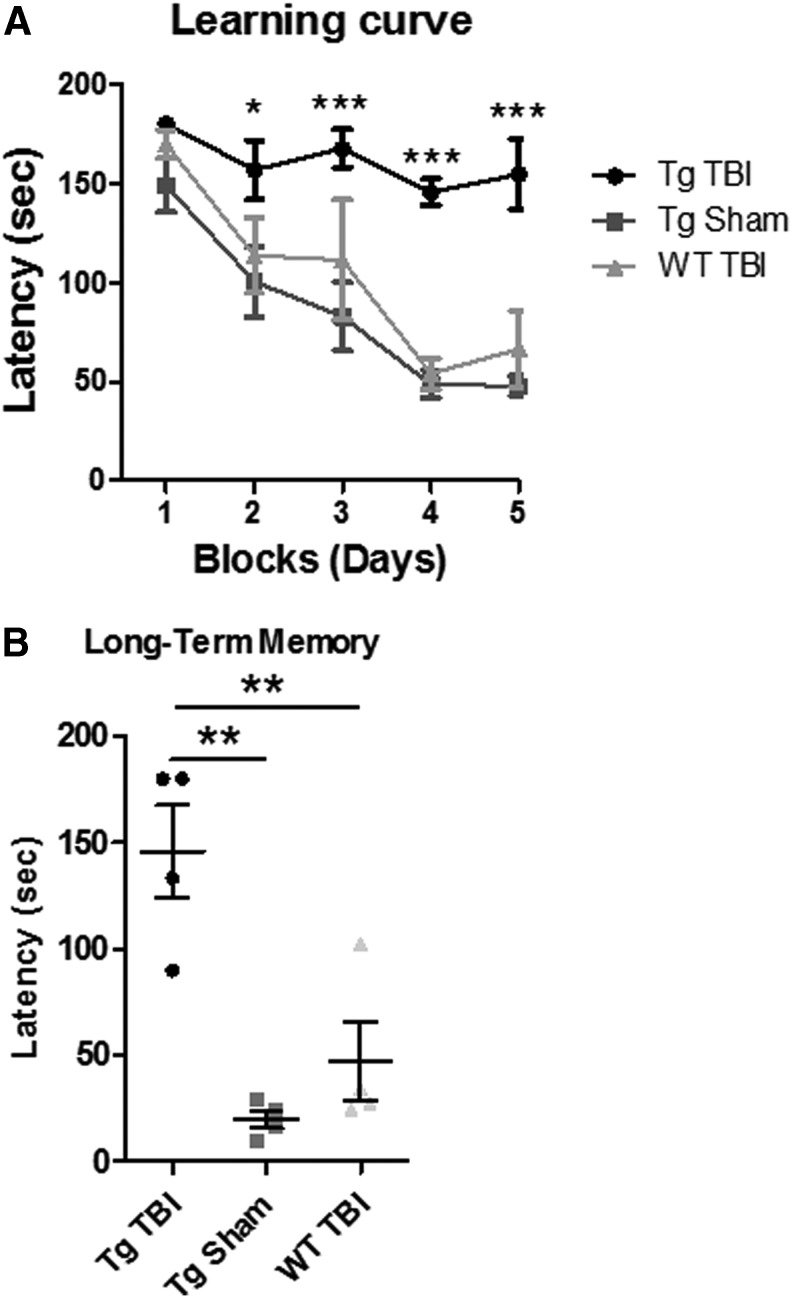
Traumatic brain injury (TBI) promotes behavior impairment in transgenic tau mice in the chronic stage of injury. Barnes maze test was performed on P301S TBI, P301S sham, and wild-type (WT) littermate TBI mice six months after controlled cortical impact injury. The P301S TBI mice exhibited attenuated learning and memory capabilities observed by the learning curve (**A**) and long term memory assessment (**B**) *p < 0.05, **p < 0.01, ***p < 0.001.

To evaluate the long-term effect of a single moderate to severe TBI event, we induced CCI in Tg and WT animals and analyzed them six months post-injury. The Tg sham animals were also evaluated. Animals 5.5 months after either CCI or sham surgery were analyzed in Barnes maze to evaluate spatial learning and memory. During the learning period, TBI-induced WT and Tg sham mice demonstrated their ability to learn a new task by a quicker latency to the escape hole over time; however, Tg TBI-induced mice took longer compared with the control animals to learn the task (*p* < 0.05, *p* < 0.001**)** (Fig. 4A).

At seven days later, long-term memory was examined, and Tg TBI-induced animals revealed significant impaired memory abilities in comparison with controls (*p* < 0.01) (Fig. 4B). Thus, Tg TBI mice posited impaired learning and memory several months after the CCI induction.

**FIG. 5.**
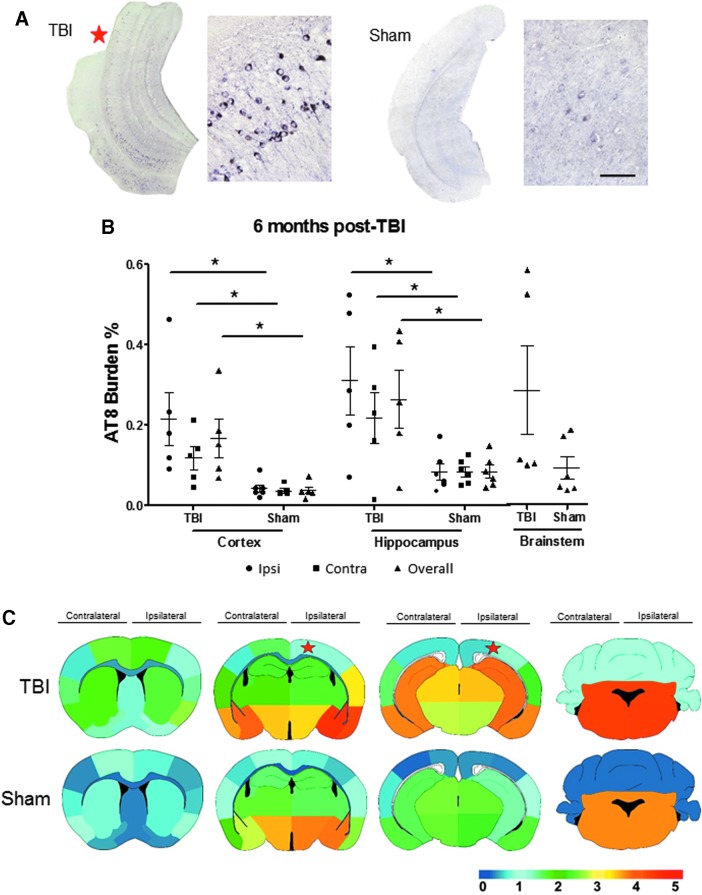
Robust tau pathology six months after traumatic brain injury (TBI). (**A**) Example AT8 microscope images illustrate substantial tau pathology in TBI mice six months after injury compared with age-matched sham mice. (**B**) The AT8 burden quantification shows a significant overall increase of tau deposition in TBI mice compared with sham mice in the ipsilateral (Ipsi), contralateral (Contra), and overall cortex and hippocampal area. Statistics for brainstem were calculated by Mann-Whitney test, while all other were performed by Student *t* test. (**C**) The AT8 burden scores exhibited in a spatial heat map of four rostrocaudal areas in TBI versus sham mice. Heat map scale: lowest (0) to highest (5) score. Star denotes the estimated placement of impact. Scale bar: 100 μm. *p < 0.05.

Then, we evaluated tau burden and created a heat map of tau deposition six months post-TBI. Tau pathology was more dramatic in the TBI-induced mice in comparison with age-matched sham controls as observed in representative immunohistochemical images. As observed in Figure 5A, NFTs-like structures were seen profusely throughout the entorhinal cortex and hippocampus both in the superficial molecular and deep polymorphic layers with intense staining in the pyramidal cells in CA1. The Tg TBI mice displayed a robust tau burden in the isocortex and throughout the hippocampal formation as shown previously.^51^ AT8 burden quantifications revealed significant differences between TBI and sham mice in cortical and hippocampal areas (Ipsi, Contra, and overall Ctx; Ipsi, Contra, and overall Hp *p* < 0.05) (Fig. 5B).

The tau heat map shows the intrinsic tau spreading mosaic of the Tg model of the sham group that seems to be maintained in the TBI-induced mice albeit with augmented dispersion and increased tau levels (Fig. 5C). Areas that exhibited the greatest tau burden scores at this late stage time point were the amygdaloid nucleus and piriform/entorhinal cortex as well as caudally in the brainstem (pons-medulla).

### TBI-induced mice demonstrate tau deposition altered tropism

**FIG. 6.**
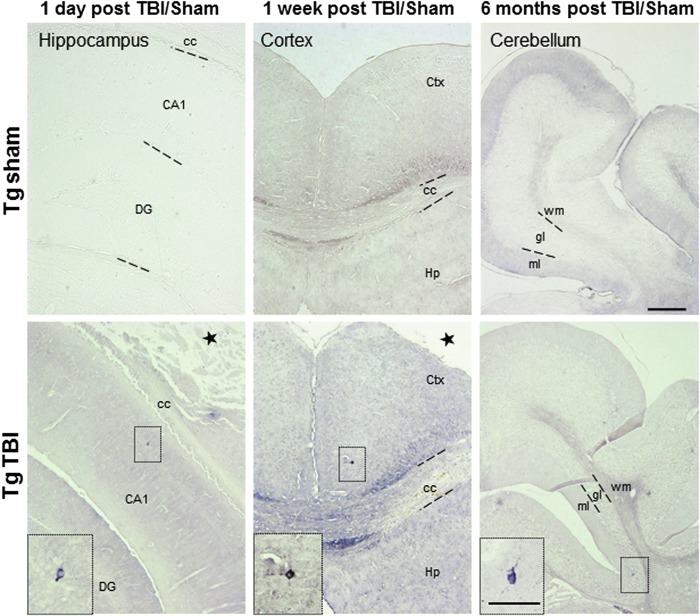
Altered tropism of tau deposition after traumatic brain injury (TBI). Representative figures of pathological tau deposition as evaluated by AT8 immunohistochemistry in hippocampus, cortex, and cerebellar lobes in mice six months after TBI. Age-matched P301S sham animals revealed very low detectability using AT8 at the different times analyzed. Scale bar: 100 μm; insets: 30 μm. CA1, cornu ammonis 1 area; cc, corpus callosum; DG, dentate gyrus; Ctx, cortex; Hp, hippocampus; ml, molecular layer, gl, granular layer; wm, white matter.

To investigate whether the increase of tau burden produced by TBI is just because of an increase in the endogenous tau production or the induction of tau aggregation in areas where tau usually do not deposit, we analyzed at different time points the brain areas where the P301S mice typically do not have tau deposition. Images taken one day post-TBI using the AT8 antibody indicate the existence of tau hyperphosphorylation in the CA1 region of the Ipsi Hp, infrequent at this age in non-induced P301S mice (Fig. 6, left panel).

The altered tropism of tau aggregation after TBI is also observed one week post-TBI in the cortical area, close to the retrosplenial cortex (Fig. 6, middle panel), as well as in the cerebellum six months after TBI (Fig. 6, right panel). It is noteworthy that, along with the total aggregated tau burden assessment performed in Tg TBI and Tg sham mice, no AT8 immunopositive tau was detected in WT TBI-induced animals even six months post-injury (data not shown).

**FIG. 7.**
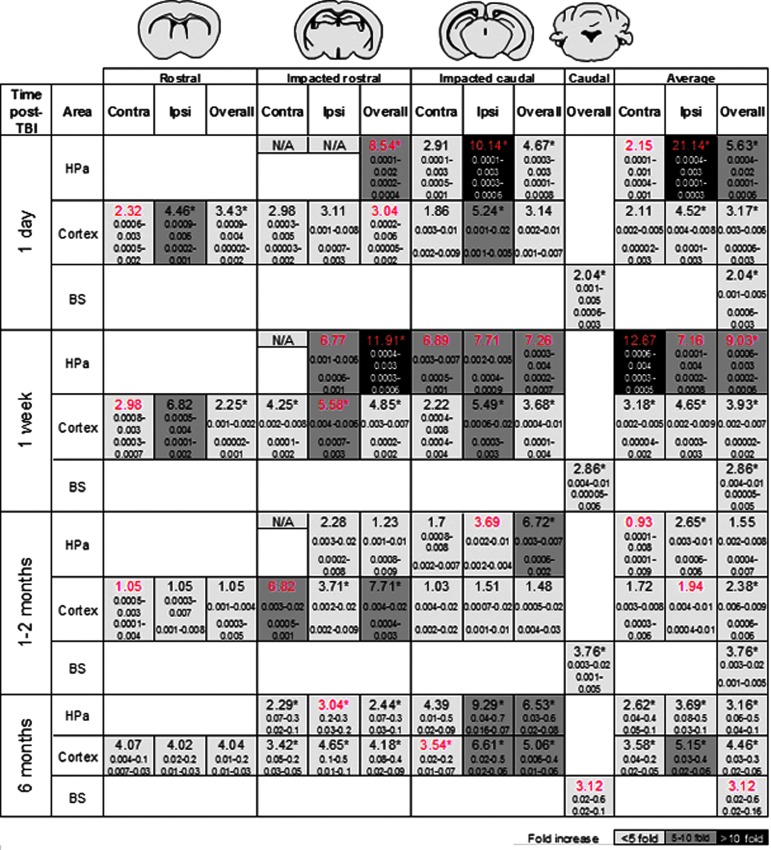
Traumatic brain injury **(**TBI)-induced tau aggregates fold-change summary. The AT8 burden fold-change is represented in the overall, ipsilateral (Ipsi) and contralateral (Contra) side of impact in the hippocampal area (HPa), cortex/amygdala, and brainstem (BS) in relation to the respective brain areas rostrocaudally to the impacted side in TBI versus sham induced mice. The colored scale exhibits the lowest to highest fold change compared with sham brains. The fold-change values displayed in red were analyzed by Mann-Whitney test while all other data analysis was performed by Student *t* test. The values below the fold change display the lower to upper 95% confidence interval of the mean of the TBI (top) and sham (bottom) groups. *p < 0.05.

Figure 7 summarizes the fold-change for aggregated tau burden in brain between TBI- and sham-induced mice after one day, one week, 1–2 months, and six months after the surgery. This summary of tau distribution encompasses the Ctx, Hp, and BS in relation to the impacted area (overall, Ipsi, and Contra). In general, there is a significant fold-change response in the Ctx, Hp, and BS of the TBI mice compared with sham mice at the various assessed time points with a majority of the regions displaying significant increase overall and on the Ipsi side of impact.

The largest fold-change (21.14) is seen after one day post-CCI impact in the ipsilateral Hp, followed by the more caudally impacted area in the Hp (10.14 fold-change). After one week, the largest fold change difference that occurred after CCI was on the contralateral side of impact for the Hp followed by the Ctx compared with their respective sham group. Thus, tau aggregate formation exacerbates one day after TBI and spreads throughout the brain significantly, even to the contralateral side of impact, one week after. Although differences in tau burden fold-change decrease at one and six months post-TBI, these alterations are kept at 3–8 fold-increase in the Hp and Ctx over time. On the other hand, one area with the highest endogenous tau deposition in this animal model, the BS, is the least affected by TBI (between 2–3 fold-increase).

## Discussion

TBI has been reported to be an important risk factor for several tauopathies.^1–3^ Experimental models and human cases have shown formation of tau aggregates after a moderate to severe TBI; however, the exact linkage to deposition of pathological tau and disease remains to be elucidated.^19^ In this study, young P301S mice subjected to TBI were analyzed at short and long time points after the impact, and brains were assessed for tau aggregation and spreading.

We have determined that TBI induction is able to increase tau burden as early as one day post-TBI in the cortex, amygdala, hippocampal area, and brainstem with robust deposition on the ipsilateral side of the impact. One week after TBI, induced mice exhibited a robust deposition on the contralateral side of the brain compared with sham animals. Increased levels of tau after TBI can translate to aggravated behavior and clinical symptoms, as observed six months after injury.

Overall, we have demonstrated that TBI intensifies tau pathology compared with sham transgenic mice in different brain regions, especially those close to the impacted area, showing a peak a few days later. Levels of aggregated tau have been found augmented in 6-month-old P301L mice one day after induction of CCI, thus supporting our results.^56^ Other moderate-severe TBI models, such as fluid percussion injury,^57^ Feeney's weight drop,^58^ and blast injury,^59^ have reported ptau augmentation in mouse and rat models. In addition, induction of CCI in AD mouse models overexpressing amyloid beta, such as 3xTgAD and Tg2576, have also demonstrated increased ptau.^60,61^ None of the previous studies, however, have shown the temporal distribution of ptau and tau aggregates after the initial insult and the spatial spreading of the misfolded tau from the original impacted area.

The results obtained in our study support the idea that TBI is a risk factor for tauopathies and provide novel insight into the induction of ptau pathology after TBI. Our study has some limitations, however. The PS19 (P301S mutation) model overexpresses five-fold the human mutant tau protein under the prion promoter, which may differentially alter expression of MAPT in different brain regions leading to ectopic endogenous expression. In addition, the human version of tau in this animal is 4R1N, which may influence the propensity of tau to aggregate after TBI, the type of aggregates that are formed, or even their tropism.

Nevertheless, the PS19 model has been proposed as a good alternative to study acceleration of the disease and tau transmission.^62^ The use of humanized tau models expressing WT human tau may help to overcome these limitations. The use of animals conserving the human 3R/4R ratio will also be beneficial to determine the effect of TBI, specifically for AD.

The TBI-induced mice had elevated levels of ptau compared with sham mice in the ventral isocortex, in particular amygdaloid nucleus, piriform and entorhinal cortices, indicating an innate tau progression associated with the regular tau pattern deposition in the P301S mouse model.^45^ These areas are also the first ones affected by tau pathology in early stages of AD.^6^

When comparing the ipsilateral and contralateral sides of impact, it revealed higher tau burden on the impacted hemisphere, indicating the direct association between the TBI and increased aggregation of tau. Tau deposition was seen in the ipsilateral hippocampus, just below the directly impacted area, as well as less common areas that would not normally have tau deposition in the naïve Tg mouse such as the cingulate gyrus, auditory and vision cortices, corpus callosum, and cerebellum. In fact, cerebellar tau aggregation has been noted in the P301S mouse model but usually occurs at a later age than assessed here, similar to the tau pathology observed in AD patients.^63,64^ Therefore, TBI not only increases tau deposition nearby the impacted area, but also induces tau aggregation in distant areas that are not usually affected in animals not subjected to TBI.

The uncommon, altered tropism of tau phosphorylation observed in TBI-induced animals could be because of different mechanisms such as contrecoup, where the ventral portion of the brain could collide on the posterior skull or by acceleration and deceleration forces. Consequently, the contrecoup could be playing a role in tau aggregation because the ventral cortex continuously displayed elevated tau burden after TBI from the secondary brain injury inside the skull. Our results posit that TBI can elicit the formation of early tau aggregates exacerbating the ipsilateral side of impact while pathological tau propagates to other brain areas increasing its burden along neuronal connections causing widespread distribution of tau pathology over time.

Another possibility for the increase of tau aggregation in areas distant from the impacted region is the spreading of pathologic tau through neuronal connections. It is already accepted that tau aggregates can propagate through functionally connected brain regions after the exogenous addition of seeding-competent material, whether disease-associated brain homogenate or recombinant tau aggregates.^28,34–36,65^ In addition, the Tg4510 mouse model, which expresses human mutant P301L tau in the entorhinal cortex, is able to develop full brain tau pathology because of the spreading of tau pathology from the initial expression brain region to the hippocampus, neocortex, and amygdala over time, indicating the propagation of misfolded tau through interconnected regions as a mechanism for disease progression.^66^

Our results indicate that tau propagation seems to occur by neuronally connected areas after TBI in a stereotypical and temporal pattern and less likely by spatial proximity, because we do not see an increase in tau aggregation in the damaged area over time—mostly in nearby proximity. As observed in the heat maps, the brain regions most affected are those with direct projections to or from the impacted area including the entorhinal cortex, hippocampal area, amygdala, brainstem, piriform and rhinal cortices, hypothalamus, and septal nucleus.

It is worth to mention that this study does not validate spreading of tau pathology by a prion-like manner after TBI. Seeding capability of the aggregates formed after TBI has not been tested by techniques such as protein misfolding cyclic amplification or bioassays; therefore, TBI-induced misfolded tau may or may not be seeding-competent.^67^ The spreading observed here may be because of regular transport through the synapses or induction of tau hyperphosphorylation in different regions from a contrecoup or associated inflammation maintained over time.

The alternative spatial aggregation pattern could also be determined by the formation of new tau conformations after TBI. Tau can aggregate acquiring different conformations, also termed strains.^67–69^ Inoculation of brain homogenate from different tauopathies into mice induced comparable tau inclusions to that of the exogenous brain extract, thereby stably maintaining the specific strain assembly or conformation reminiscent of the initial pathology.^34,35^ Eighteen tau strains have been characterized and, when inoculated in P301S mice, they trigger strain-specific intracellular pathology in specific brain regions, spreading, and toxicity.^68^

It is yet to be determined whether TBI is responsible for generating tau strains different from those regularly expressed in the mouse model and whether this phenomenon might be responsible for spreading the pathology to brain regions that are not commonly affected. In fact, AT8 antibody was utilized for assessing tau pathology after TBI, and it is able to recognize both pre-tangle and tangle pathology in brain. Therefore, utilization of conformational specific antibodies of tau could be used to determine the exact pathological tau species and their location in brain after TBI.

In addition, the increase in tau hyperphosphorylation may have a potential pathophysiological role in the progression of the disease or be simply a consequence of other effects that TBI could be causing, such as a modification in the kinase/phosphatase expression and activity by which elevation of ptau could be initiated as a response to a general damage in the brain.

An ancillary factor in regard to the sequential pattern of tau deposition is a different brain regional vulnerability and the existence of a variety of neuronal types that can get affected differently to tau pathology progression. The areas chiefly affected by tangles in AD, according to Braak staging, are those involved in higher-order cognitive functions, areas vulnerable in aging, as well as those that take the longest time for reaching maturity during development.^2,70^ The TBI could also generate a hostile brain microenvironment facilitating tau aggregation and propagation. After a TBI event, this response can therefore be intensified, stimulating early disease-affiliated tau aggregates leading to disease onset and irreversible progression.

Overall, our results propose an increasing progression of tau aggregation after one single moderate to severe TBI, indicating that TBI accelerates tau hyperphosphorylation and aggregation in a time- and spatial-dependent manner. The formation of tau misfolded aggregates after the initial TBI may further template the aggregation of additional pathogenic units, participating in the formation of primordial protein misfolded seeds that will template pathological features leading to AD, CTE, or other tauopathies. It remains to be identified the conformation and molecular properties of pathological tau induced by TBI events and what features these *de novo* generated aggregates have compared with tau deposition found in sporadic tauopathies.

## Supplementary Material

Supplemental data
