# Supplementary material for: Traumatic Brain Injury Induces Tau Aggregation and Spreading
Source: J Neurotrauma. 2019 Dec 11;37(1):80–92. doi: 10.1089/neu.2018.6348 (PMC6921297; doi:10.1089/neu.2018.6348)
Supplement: Supplemental data [file Supp_FigureS1.pdf]

Supplementary Data

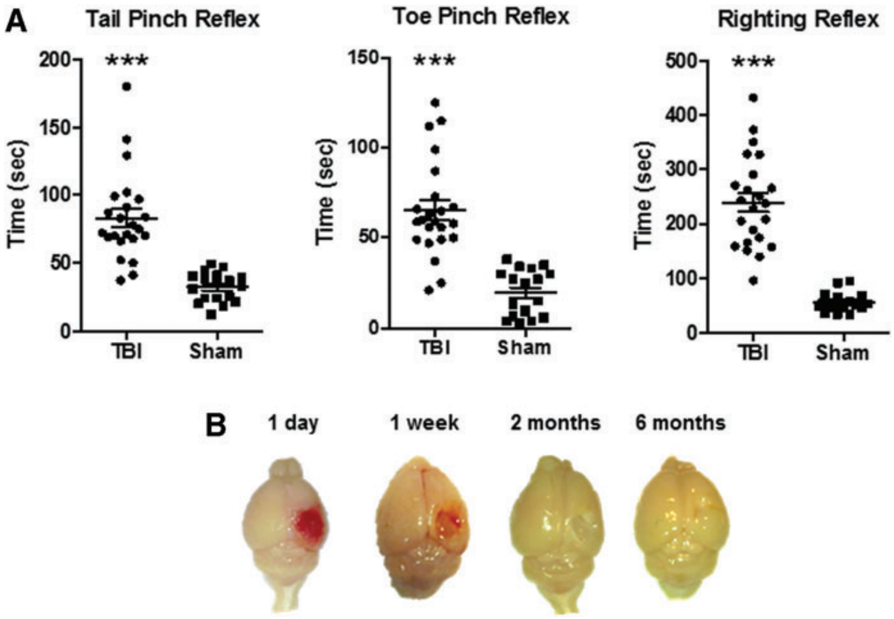

SUPPLEMENTARY FIG. S1. Traumatic brain injury (TBI)-associated impaired function and brain damage. (**A**) Mice were tested for somatosensory functions immediately after controlled cortical impact (CCI) or sham surgery by Mann-Whitney test (***$p < 0.001$). (**B**) Representative images of brain damage after CCI over the right parietal cortex in mice after one day, one week, two months, and six months post-operation are shown. Hematoma and edema are indicated transiently after impact, thereby forming a collapsed 1 mm deformation in brain at later stages.
